# Ultrasound-Assisted Extraction of Paeonol from Moutan Cortex: Purification and Component Identification of Extract

**DOI:** 10.3390/molecules29030622

**Published:** 2024-01-28

**Authors:** Ling Meng, Yan Chen, Zhenjia Zheng, Lei Wang, Yahui Xu, Xiujun Li, Zhijian Xiao, Zheng Tang, Zhaosheng Wang

**Affiliations:** 1 Key Laboratory of Food Processing Technology and Quality Control in Shandong Province, College of Food Science and Engineering, Shandong Agricultural University, Taian 271018, China; mengling202309@163.com (L.M.); chenyan70918@163.com (Y.C.); zhengzhenjia@sdau.edu.cn (Z.Z.); wl1842350751@163.com (L.W.); xyh19806105326@163.com (Y.X.); 15615687016@163.com (X.L.); 2 Shandong Wake Fresh Food Technology Co., Ltd., Taian 271400, China; 15s-euiewb5y4r@dingtalk.com (Z.X.); wkxyanjiu001@dingtalk.com (Z.T.)

**Keywords:** Moutan Cortex, paeonol, extraction, purification, macroporous resin, UPLC-Q-TOF-MS/MS

## Abstract

Moutan Cortex (MC) is a traditional Chinese medicine that contains abundant medicinal components, such as paeonol, paeoniflorin, etc. Paeonol is the main active component of MC. In this study, paeonol was extracted from MC through an ultrasound-assisted extraction process, which is based on single-factor experiments and response surface methodology (RSM). Subsequently, eight macroporous resins of different properties were used to purify paeonol from MC. The main components of the purified extract were identified by ultra-performance liquid chromatography–quadrupole–time of flight–mass spectrometry (UPLC-Q-TOF-MS/MS). The results indicate the optimal parameters are as follows: liquid-to-material ratio 21:1 mL/g, ethanol concentration 62%, ultrasonic time 31 min, ultrasonic temperature 36 °C, ultrasonic power 420 W. Under these extraction conditions, the actual yield of paeonol was 14.01 mg/g. Among the eight tested macroporous resins, HPD-300 macroporous resin was verified to possess the highest adsorption and desorption qualities. The content of paeonol increased from 6.93% (crude extract) to 41.40% (purified extract) after the HPD-300 macroporous resin treatment. A total of five major phenolic compounds and two principal monoterpene glycosides were characterized by comparison with reference compounds. These findings will make a contribution to the isolation and utilization of the active components from MC.

## 1. Introduction

The peony (*Paeonia suffruticosa* Andr.) is a perennial deciduous shrub in the genus Paeonia of the buttercup family. It is native to Asia, Europe, and North America, and is widely cultivated in China and Japan [1]. It not only has good ornamental value, but it also has medicinal value. Moutan Cortex (MC), as the main medicinal part, is the dry root bark of the peony, and is one of the most important herbs in traditional Chinese medicine [2]. MC was first published in the *Shennong’s Herbal Classic of Materia Medica* [3] and performs a variety of medicinal functions, such as anti-inflammatory [4], antioxidant [5], anti-bacterial, anti-cancer [6], and anti-diabetes [7]. There are many components isolated from MC, mainly including phenols and phenolic glycosides, monoterpenes, and their glycosides, sterols, flavonoids, coumarins, polysaccharides, and organic acids, etc. It was revealed that paeonol and paeoniflorin are considered as the staple bioactive components of MC [8].

Paeonol has been regarded as an important index to evaluate the quality of MC in the *Pharmacopoeia of People’s Republic of China*. Pharmacological evidence demonstrates that paeonol has bacteriostatic, anti-inflammatory [9], antioxidant [10], anti-tumor [11], antiatherosclerosis [12], neuroprotective [13], and anti-diabetic effects [14]. There are abundant resources of MC in China, but most of them are not fully utilized, except for some of them being exported as medicinal materials or used for prescriptions, resulting in a waste of resources. The price of natural paeonol is several times that of chemical synthetic products, and the demand exceeds the supply in the international market [15]. In view of the beneficial pharmacological activities and growing market demand for paeonol, it is of interest to develop a simple and effective process for the extraction and purification of paeonol from MC.

Conventional extraction procedures, such as steam distillation extraction [16], decoction extraction, and organic solvent extraction, have been applied to isolate paeonol from MC, but these methods suffer from deficiencies including low extraction rates and long extraction durations. These shortcomings have led to the consideration of emerging technologies such as supercritical fluid extraction [17] and ultrasound [18]. Although supercritical fluid extraction has less toxic and high extraction efficiency properties [19], it requires expensive equipment and is not suitable for mass production. Ultrasound-assisted extraction is a green technology that has been applied for phenolic compound extraction because of its low cost, low time consumption, high yield, and ability to minimize the damage to the extracted components [20,21]. Ethanol, as a green solvent, is a promising solvent that has been used for extracting bioactive compounds due to its many advantages, including operational safety, high purity, and renewability. Additionally, it can reduce greenhouse gas emissions [22]. Thus, ultrasound-assisted ethanol extraction is a suitable technique for the extraction of paeonol. Natural phenolic compounds are usually purified by resin adsorption [23], recrystallisation, column chromatography [24], high-speed counter-current chromatography [25], preparative high-performance liquid chromatography [26], etc. At present, the purification methods of paeonol are mainly focused on recrystallization and high-speed countercurrent chromatography [27]. Recrystallisation is a traditional purification method with high solvent requirements. HSCCC is a classic separation technique based on the liquid–liquid partition principle, which has been used in the separation of natural active compounds [28,29]. However, it is subject to special demands on technique, resulting in longer operating recycling, a large amount organic solvent consumption, and high costs [30]. Macroporous adsorption resin is a type of organic polymeric absorbent with physicochemical properties that are inexpensive, readily available, and have excellent selectivity [23]. Comparatively, the macroporous resin adsorption method seems to be the most suitable method due to its low cost, low solvent consumption, and environment friendliness [23,31]. At present, it has been widely applied for the purification of bioactive components, such as flavonoid [32,33,34], polyphenol [35,36,37,38,39,40], and monoterpene glycoside [41,42,43]; however, there is no report on using macroporous adsorption resin to purify paeonol from MC.

Research methods on the chemical constituent analysis of MC has been extensively reported, and includes liquid chromatography–diode array detector–electrospray mass spectrometry (LC-DAD-ESIMS) [44], high-performance liquid chromatography–diode array detector (HPLC-DAD), inductively coupled plasma–mass spectrometry (ICP-MS) [45], etc. Accumulating evidence has suggested that ultra-performance liquid chromatography–quadrupole–time of flight–mass spectrometry (UPLC-Q-TOF-MS/MS) is an excellent technique for qualitative analysis of multiple components in complex mixtures due to its superior performance, high resolution, high sensitivity, fast scanning speed, and wide dynamic range, coupled with the efficient separation capability and speed of ultra-performance liquid chromatography (UPLC) [46]. In addition, there have been various applications for the screening of bioactive compounds of MC [47,48], the evaluation of quality markers [49], the chemical fingerprinting of paeonol [45], and the quantitative determination of herbal medicines [50]. However, due to the fact that the identified compounds were prepared by means of an organic solvent in these reports, there were derivatives, geometric isomers, and more than a hundred compounds [5]. This is not conducive to the qualitative analysis of the bioactive components. Up to now, there have been no reports about the identification of the bioactive components from MC extract by UPLC-Q-TOF-MS/MS.

This study was carried out with the purpose of developing a simple and environment-friendly process for the enrichment of paeonol and identifying the active composition of MC extract for further research in pharmacological activity. In this study, a crude MC extract enriched paeonol was produced by an ultrasound-assisted ethanol method. Subsequently, the purification of the MC extract was investigated using the macroporous resin absorption method. Both the extraction and the purification process were optimized. Eventually, UPLC-Q-TOF-MS/MS was used for the first time to analyze the main components of the MC extract. These findings will provide a theoretical basis for the effective development and utilization of MC resources.

## 2. Results and Discussion

### 2.1. Extraction of Paeonol from MC

#### 2.1.1. Effects of the Various Factors on the Extraction Yield of the Paeonol

**Figure 1:**
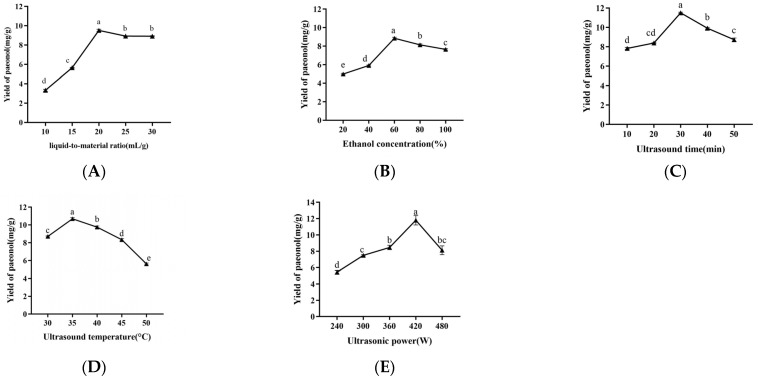
The effects of liquid-to-material ratio (**A**), ethanol concentration (**B**), ultrasonic time (**C**), ultrasonic temperature (**D**), and ultrasonic power (**E**) on the extraction yield of paeonol. The values are expressed as means ± SD (*n* = 3). Different lowercase letters (a, b, c, d, and e) in the same figure indicate statistically significant differences (*p* < 0.05).

In general, appropriately increasing the liquid-to-material ratio could dissolve the components more effectively, resulting in improving the extraction yield [51]. As exhibited in Figure 1A, the paeonol yield increased with the increasing liquid-to-material ratio, ultimately reaching a maximum of 20:1 mL/g. When the liquid-to-material ratio was greater than 20:1, the yield of paeonol slowly decreased. These results may be due to the increase of the extraction solvent making the contact area between the solid and liquid phases increase significantly, and the mechanical effect of the ultrasound increasing the cell permeability, leading to a faster diffusion ratio; thus, the yield of paeonol gradually increased. When the liquid-to-material ratio increased to a certain value, the yield of paeonol decreased. It may be that a certain proportion of the solvent had completely dissolved the paeonol, and further increasing the liquid-to-material ratio will lead to the dissolution of more impurities and affect the dissolution of paeonol [52]. Considering the factors of energy consumption and the subsequent workload, 20:1 was selected as the ideal liquid-to-material ratio in the response surface methodology (RSM) experiments.

As exhibited in Figure 1B, the yield of paeonol initially increased and then decreased as the ethanol concentration increased; the highest paeonol yield occurred at the ethanol concentration of 60%. This might be due to the fact that the polarity of the 60% ethanol was the closest to that of the paeonol, which rendered the paeonol more easily dissolvable [53]. According to the similarity and compatibility [54], when the polarity of the paeonol was similar to that of the ethanol solution, the solubility of paeonol was the highest. When the ethanol concentration was below 60%, the polarity of the ethanol solution increases, leading to decrease in the dissolution of paeonol, while when the ethanol concentration was greater than 60%, the polarity of the ethanol solution decreases, which was not conducive to the dissolution of paeonol. Accordingly, 60% was selected as the ideal ethanol concentration in the RSM experiments.

Ultrasound has the effect of cavitation and fragmentation, which can accelerate the leaching of active components and shorten the time of ethanol extraction. It was shown, as evidenced in Figure 1C, that the yield of paeonol initially increased and then decreased with the prolonging of the ultrasound time, eventually reaching a maximum at 30 min. The cavitation effect of ultrasound, to some extent, could increase the contact surface area between the solid phase and the liquid phase, resulting in an increased yield of paeonol [20,55]. However, the yield of paeonol decreased when the ultrasonic time exceeded 30 min, which may be attributed to the loss of paeonol with the evaporation of water vapor, along with the extension of the ultrasonic time [15]. Accordingly, 30 min was selected as the ideal ultrasound time in the RSM experiments.

As depicted in Figure 1D, the paeonol yield firstly increased and then decreased as the temperature raised, and the optimum values were reached when the temperature was 35 °C. Appropriately increasing the temperature accelerates the movement of the solvent and solute molecules, which favors the release of paeonol from the raw material to the solvent and promotes diffusion. However, excessive temperatures caused the paeonol to be thermally oxidized, resulting in a decline in the extraction yield [55,56]. These results indicate that the extraction temperature had a significant positive effect on the paeonol yield when it was below 35 °C. Therefore, 35 °C was appropriate for the ultrasound temperature.

As displayed in Figure 1E, different ultrasound powers ranging from 240 W to 480 W were tested. The yield of paeonol increased first and then decreased with the increase of ultrasound power, and peaked at 420 W. The result was in accordance with other reports for the extraction of phenolic compounds [53]. This might be responsible for the increase of the mass transfer ratio of paeonol with high ultrasonic power [57]. Therefore, it was determined that the ultrasonic power of 420 W should be utilized.

#### 2.1.2. Response Model Establishment and the Variance Analysis

Based on the results of the single-factor experiments, the liquid-to-material ratio (A), ethanol concentration (B), ultrasonic time (C), ultrasonic temperature (D), and ultrasonic power (E) were taken as the influencing factors of the response surface test in accordance with the design principle of the Box–Behnken central combination test (Table 1).

The paeonol yield (mg/g) of the extraction process was set as a response value of the design experiments. A Box–Behnken Design (BBD) of five factors and three levels was conducted. The results are shown in Table 2.

#### 2.1.3. Response Surface Analysis

The analysis of variance (ANOVA) of the Box–Behnken was carried out by Design-Expert 13.0 software (Stat-Ease Inc., Minneapolis, MN, USA). As shown in Table 3, the model was significant (*p* < 0.0001). The F value of the lack-of-fit item was 0.22, and the *p*-value was 0.9936 (*p* > 0.05), suggesting that the model fits well and can explain the variation in the response. The regression coefficient R^2^ of the model was 0.9633, indicating that the model could explain 96.33% of the total changes in the response values, and the value of R^2^_Adj_ was 0.9339, indicating that there was a good correlation between the equation and the selected independent variables. Moreover, the primary terms (A, B, C, D, E), the interaction terms (AE, BD, BC), and the quadratic terms (A^2^, B^2^, C^2^, D^2^, E^2^) had significant effects on the yield of paeonol (*p* < 0.05).

After regression fitting, the quadratic regression model of the response value on each factor was as follows:

Y = 12.97 + 0.67A + 0.4B + 0.29C + 0.24D + 0.37E + 0.013AB + 0.12AC + 0.048AD + 0.61AE − 0.46BC + 0.60BD − 0.073BE + 0.25CD − 0.15CE + 0.23DE − 2.3A^2^ − 2.08B^2^ − 1.43C^2^ − 1.67D^2^ − 2.55E^2^.

**Figure 2:**
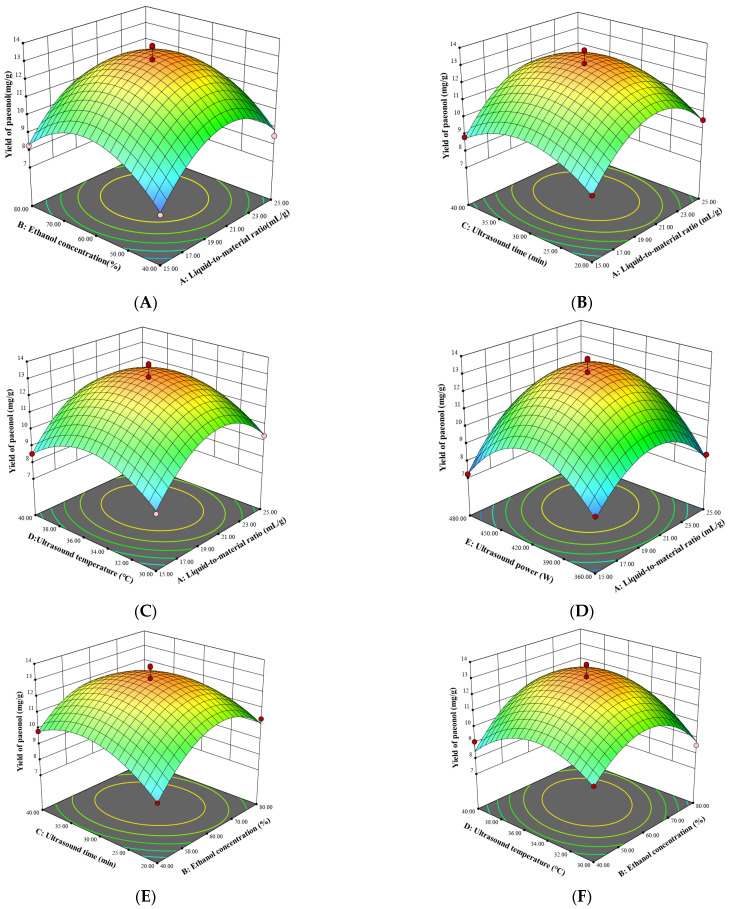

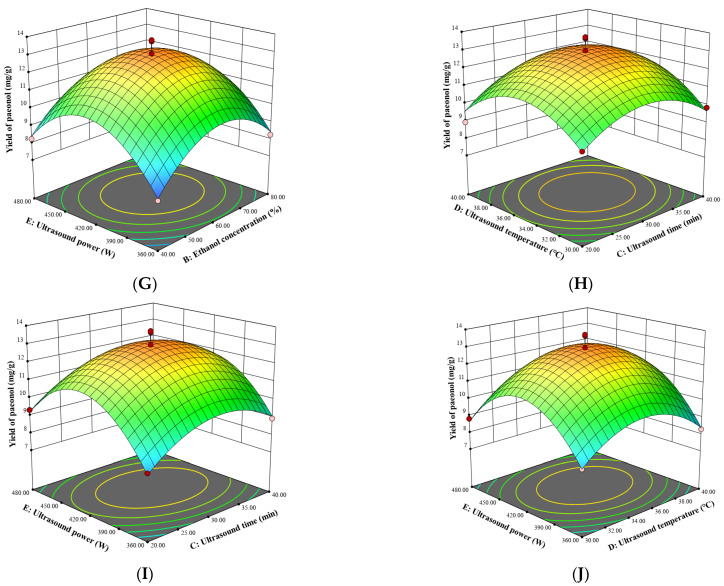
Response surface plots for interaction between various factors on the yield of paeonol. The two changed variables were liquid-to-material ratio and ethanol concentration (**A**); liquid-to-material ratio and ultrasound time (**B**); liquid-to-material ratio and ultrasound temperature (**C**); liquid-to-material ratio and ultrasound power (**D**); ethanol concentration and ultrasound time (**E**); ethanol concentration and ultrasound temperature (**F**); ethanol concentration and ultrasound power (**G**); ultrasound temperature and ultrasound time (**H**); ultrasound time and ultrasound power (**I**); and ultrasound temperature and ultrasound power (**J**).

The response surface plots of the quadratic regression equation were obtained using Design Expert 13.0 software. Figure 2 depicts the response surface plots reflected by the interaction between any two variables on the paeonol yield. In the response surface plot, the steeper the surface plot, the more obvious the interaction between the variables. As shown in Figure 2, the interaction terms (AE) had the most significant effect on the yield of paeonol, followed by BD and BC, and other interaction terms (AB, AC, AD, BE, CD, CE, DE) were not significant, which were consistent with the ANOVA in Table 3.

#### 2.1.4. Optimal Conditions and Model Validation

Based on the results above, the optimal extraction conditions of paeonol from MC were obtained: the liquid-to-material ratio was 20.81:1 mL/g, the ethanol concentration was 61.96%, the ultrasound time was 30.98 min, the ultrasound temperature was 35.52 °C, and the ultrasound power was 425.48 W. Under these conditions, the model predicted that the yield of paeonol was 13.68 mg/g. Considering the limitations of that actual operation, the process parameters were modified as follows: liquid-to-material ratio, 21:1 mL/g; ethanol concentration, 62%; ultrasonic time, 31 min; ultrasonic temperature, 36 °C, and ultrasonic power, 420 W. A verification assay was carried out based on the conditions above: the actual yield of paeonol was 14.01 mg/g, which was close to the predicted value, and the relative standard deviation was 2.36%. There was no significant difference between the two conditions, indicating that the obtained model could be used for the extraction of paeonol from MC. Under the optimal conditions, the extraction rate of paeonol reached 93.82%.

The results of the ultrasound-assisted ethanol extraction were compared with other studied extraction methods. The research of Zhuo Chen et al. [56] concluded that the total yield of paeonol and paeoniflorin from MC by ultrasound-assisted deep eutectic solvents (DESs) extraction was 9.279 mg/g under the optimal process. A study conducted by Yinshi Sun et al. [27] noted that supercritical fluid extraction was used to extract paeonol from *Cynanchum paniculatum* (Bge.) Kita. Under the optimized conditions of 15 MPa, 55 °C, 1.5 h, and repeating the process 10 times, the extraction rate of paeonol reached 72.02%. Fengli Chen et al. [58] developed a novel approach for the extraction of paeonol by microwave irradiation using an ionic liquid as the reaction medium. The yield of paeonol was 10.52 mg/g under the following optimum conditions: 20 mL/g liquid–solid ratio, 25 min microwave irradiation time, 700 W microwave irradiation power. Compared with the process parameters of the extraction methods above, ultrasound-assisted ethanol extraction is more efficient, and can obtain a higher yield of paeonol in a shorter time. It is simpler than other extraction technologies and does not require repeated extractions.

### 2.2. Purification of Paeonol from MC by Macroporous Resin Adsorption

#### 2.2.1. Screening of Macroporous Resins

**Figure 3:**
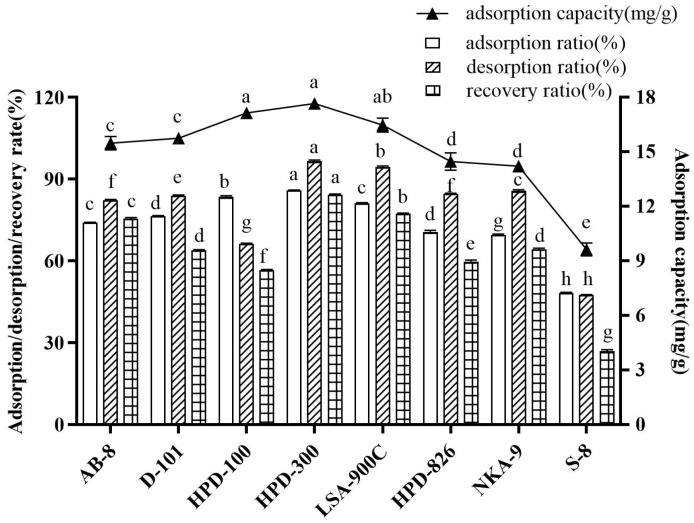
Comparison of adsorption and desorption performance of different types of macroporous resins. Notes: Those with the same letter in each column indicate no significant difference (*p* < 0.05), and those with different letters indicate significant differences (*p* < 0.05).

Generally, the adsorption properties of macroporous resin are mainly associated with the polarity and structure of the resin, such as pore size and specific surface area [38,39]. The polarity, specific surface area, and pore size of the eight resins can be derived from Table S1 (Supplementary Materials). According to the principle of similarity and compatibility, the resin of lower polarity had a better adsorption capacity for low polar and non-polar substances [35]. As shown in Figure 3, the adsorption capacity, adsorption ratio, desorption ratio, and recovery ratio of eight macroporous resins were investigated. The adsorption ratio of paeonol on HPD-300 and HPD-100 macroporous resins was prominently higher (85.77% and 83.49%, respectively) than those of other weakly polar and polar resin, indicating that hydrophobic interactions of the hydrophobic groups of paeonol may occur on the non-polar resin. The adsorption capacity of HPD-300 macroporous resin was slightly higher than that of HPD-100 resin, which was attributed to the larger surface area of HPD-300 macroporous resin providing more binding sites. Furthermore, the desorption ability of HPD-300 was significantly higher than that of HPD-100, indicating that paeonol was more easily desorbed from the resin with a large specific surface area and pore size [15]. The results are consistent with the findings reported by previous research [23]. Thus, the HPD-300 macroporous resin was selected to purify paeonol from MC, followed by the optimization of the purification condition.

#### 2.2.2. Static Adsorption and Desorption Tests

**Figure 4:**
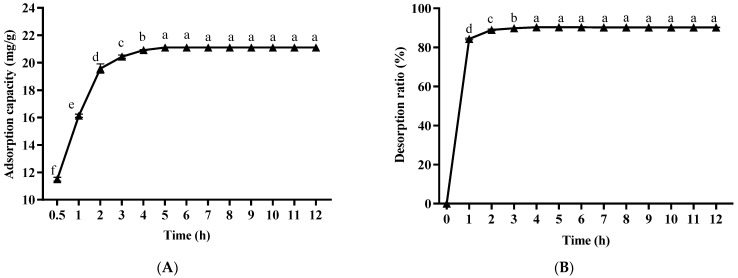
Kinetic curve of static adsorption (**A**) and desorption (**B**) of paeonol on HPD-300 macroporous resin. Lowercase letters indicate significant differences (*p* < 0.05).

The static adsorption kinetics curve of paeonol on HPD-300 macroporous resin is shown in Figure 4A. When the adsorption time was within 0–5 h, the adsorption ratio of the HPD-300 macroporous resin on paeonol increased with the extension of time, and the adsorption ratio of paeonol was the largest at 5 h. There was no significant difference in the adsorption ratio of HPD-300 macroporous resin on paeonol after 5 h (*p* > 0.05), which indicates that the adsorption equilibrium was reached at 5 h. The adsorption ratio was the highest at 5 h. Therefore, the static adsorption time of HPD-300 macroporous resin on paeonol should be around 5 h. As shown in Figure 4B, the desorption ratio of paeonol in the eluate increased first and then tended to be stable with the increase of time, and there was no significant difference in the desorption ratio when it reached 4 h (*p* > 0.05). It can be concluded that the desorption equilibrium was reached at 4 h. Hence, the static desorption time should be about 4 h.

**Figure 5:**
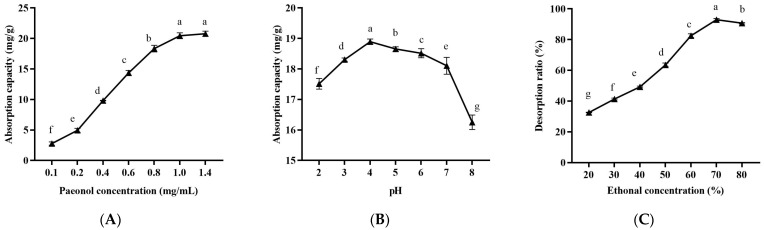
Effects of initial paeonol concentration (**A**), sample pH value (**B**), and ethanol concentration (**C**) on the adsorption capacity and desorption ratio of HPD-300 macroporous resin. Lowercase letters indicate significant differences (*p* < 0.05).

As shown in Figure 5, the effect of the initial paeonol concentration, pH value, and ethanol concentration on the static absorption and desorption of HPD-300 macroporous resin were studied. The influence of the initial paeonol concentration on static absorption is shown in Figure 5A. The adsorption capacity rose as the sample concentration increased when the paeonol concentration was less than 1.0 mg/mL, which was due to the fact that when the paeonol concentration was low, the number of active sites associated with paeonol increased as the sample concentration rose [59]. When the concentration of paeonol exceeded 1 mg/mL, the adsorption capacity no longer increased. This might be responsible for high concentrations of paeonol causing the resin to be plugged [60]. Taking this into account, the optimal concentration was determined to be 1.0 mg/mL. As shown in Figure 5B, the adsorption capacity of paeonol under a weak acid condition was higher than that under a neutral condition, and the maximum value was reached at pH 4. When the pH was higher than 4, the adsorption capacity decreased with the increase of pH, which may be attributed to the dissociation of the phenolic hydroxyl group to form H^+^ and the corresponding anions at a higher pH, which made them adsorb on the HPD-300 macroporous resin through electrostatic interactions and reduced the affinity of adsorption [31]. This result was similar to a previous report that in high pH solutions, phenolic components are not susceptible to being absorbed by the resin due to the ionization reaction [36]. Therefore, the pH value of the loading solution was set at about 4 in the following ratio experiments. In order to choose the appropriate desorption solution, elution tests were performed using different concentrations of ethanol solutions. As exhibited in Figure 5C, the desorption ratio increased significantly with the increasing ethanol concentration and reached the maximum when the ethanol concentration reached 70%. It was suggested that high concentration of the ethanol solution promoted the desorption of paeonol from MC by the HPD-300 macroporous resin. Due to the fact that ethanol reduced the polarity of water and promoted the desorption of paeonol arose from the disruption of the hydrogen bond between the paeonol and the macroporous resin, paeonol was continuously eluted with increasing ethanol concentration [31]. This result also indicates that 70% ethanol was most closely related to the paeonol’s polarity. Hence, a 70% ethanol solution was considered in the subsequent experiments.

#### 2.2.3. Dynamic Adsorption and Desorption Tests

**Figure 6:**
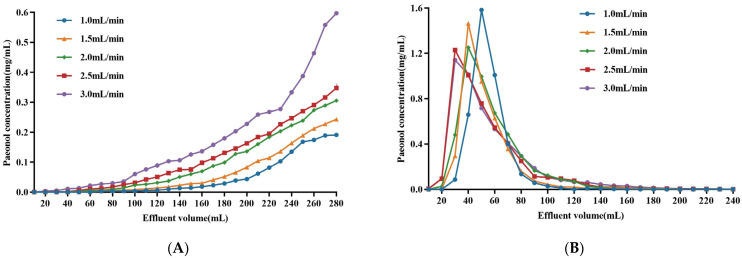
Effect of the sample solution flow rate on the adsorption of HPD-300 macroporous resin (**A**) and effect of the eluent flow rate on the desorption of HPD-300 macroporous resin (**B**).

The flow rate is the main factor affecting the adsorption ratio of resin in the dynamic adsorption process. When the paeonol concentration in the effluent reaches 1/10 of the paeonol concentration in the loading solution, it is considered to be the leakage point. When the leakage point occurs, the loading is stopped, and it is considered to be the best loading volume [34]. Figure 6 shows the effect of different loading flow rates on paeonol adsorption by HPD-300 macroporous resin. As seen in Figure 6A, the lower the loading flow rate, the later the leakage point appeared, which indicates that a low loading flow rate was more conducive to the adsorption of paeonol from MC with HPD-300 macroporous resin. As the loading flow rate increased, the concentration of paeonol increased. When the flow rate was 2.5 mL/min and 3.0 mL/min, the paeonol was not fully adsorbed by the inner surface of the resin and flowed out of the column with the permeate. Additionally, the leakage point appeared in advance, leading to poor absorption. Although the low flow rate was beneficial to improve paeonol adsorption by HPD-300 macroporous resin, the delay of the leakage point would cause low production efficiency. After a comprehensive analysis, the optimal loading flow rate was identified as 2.0 mL/min. Under this condition, the loading volume was 180 mL.

As can be seen in Figure 6B, different volumes of 70% aqueous ethanol were used to study the effect of the elution flow rate on the desorption of HPD-300 macroporous resin. It was obvious that the faster the elution flow rate, the earlier the peak time of the dynamic curve and the more obvious the trailing. It was clear that the desorption curves showed narrow and sharp outlines without the trailing phenomenon at flow rates of 1.0 and 1.5 mL/min. The concentration of paeonol in the effluent was larger, which meant that the elution effect was better. The peak shape was relatively wide, the tailing phenomenon was significant at the higher flow rate, and the concentration of paeonol in the effluent was low. These results might be attributed to the fact that the lower flow rate facilitated the interactions between the eluent and the paeonol in the resin, and a higher flow rate failed to sufficiently dissolve paeonol in the resin column [30]. The elution time and the amount of ethanol were taken into account: a 1.5 mL/min flow rate was adopted for desorption, and the eluting volume was set as 140 mL.

Therefore, the optimal purification conditions of paeonol from MC using HPD-300 macroporous resin were as follows: the sample concentration, 1.0 mg/mL; the sample pH, 4; the ethanol concentration of the eluent, 70%; the loading flow rate, 2.0 mL/min; the sample volume, 180 mL; the elution flow rate, 1.5 mL/min; the elution volume, 140 mL. Under these conditions, the purity of the paeonol in the extract increased from 6.93% to 41.40%.

### 2.3. Compositional Analysis of MC Extract

**Figure 7:**
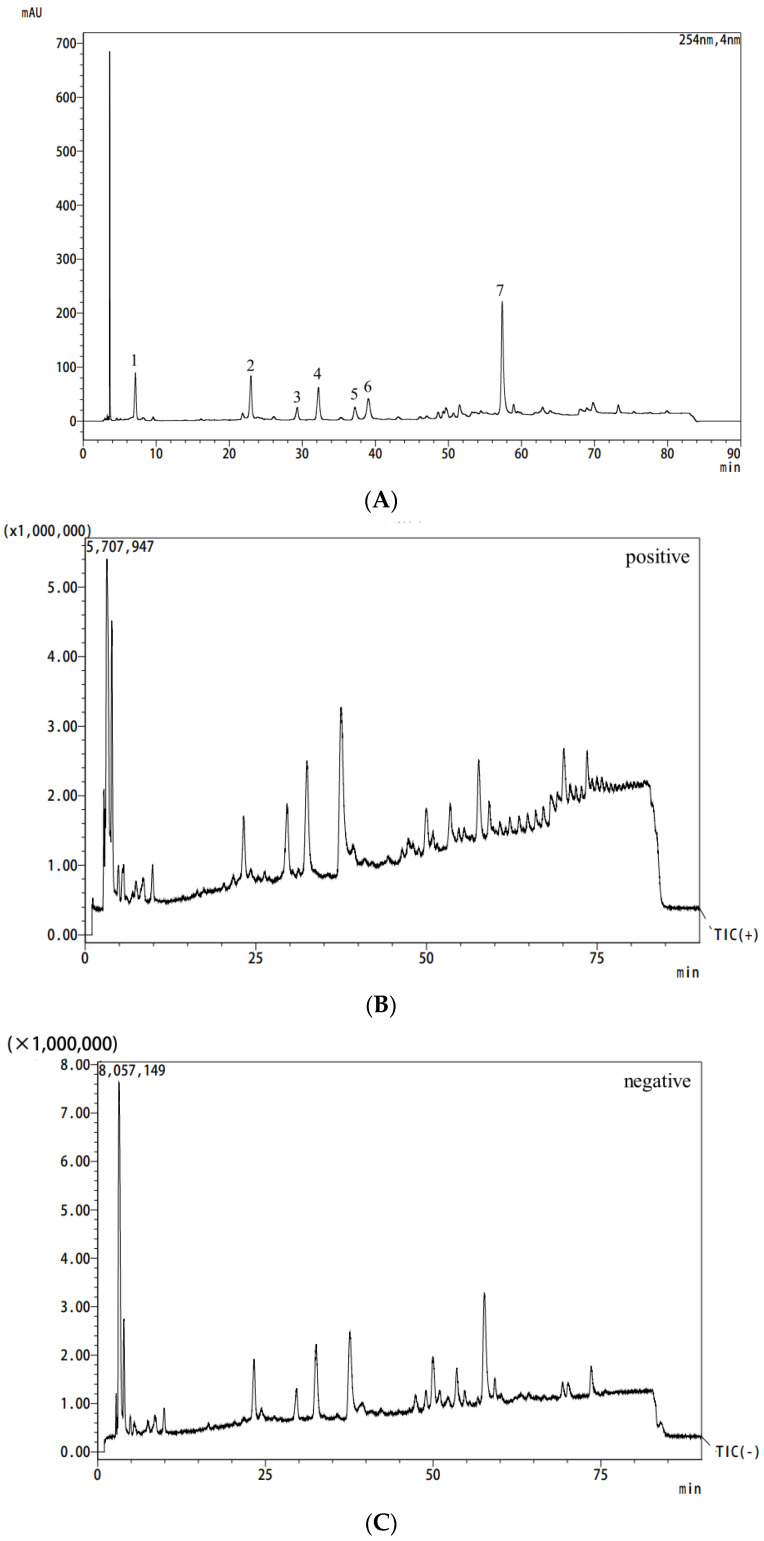
Analysis of MC extract by HPLC/MS: total ion current plots in liquid chromatograph (**A**), positive ion mode (**B**), and negative ion mode (**C**). Notes: Numbers 1–7 in Figure (**A**) indicate gallic acid (1), oxypaeoniflorin (2), paeonolide (3), paeonol (4), paeoniflorin (5), ethyl gallate (6), and pentagalloylglucose (7), respectively.

The chemical compositions of the MC extract were analyzed by UPLC-Q-TOF-MS/MS. Good chromatographic resolution was obtained by gradient elution with 0.1% (*v*/*v*) aqueous formic acid water and methanol as the mobile phase. Since the main chemical components in MC are phenols and phenolic glycosides, monoterpenes and their glycosides, and other compounds with great differences in UV absorptions, the wavelength of 254 nm, which has the most chromatographic peaks, was chosen. In this experiment, positive and negative ion modes were employed for full-scan detection, and the total ion chromatograms, HPLC chromatograms (λ = 254 nm), in positive and negative ion modes are shown in Figure 7. Seven chemical constituents were preliminarily confirmed using the method of quasi-molecular ion peak and characteristic fragment ions, combined with the discovered chemical components of the MC extract, a comprehensive analysis of the retention time, relative molecular mass, fragment ion information of each compound, and comparing with the literature data, which included gallic acid, paeonolide, paeonol, and other compounds of the MC extract. The results of the UPLC-Q-TOF-MS/MS analysis for each peak are shown in Table 4.

**Figure 8:**
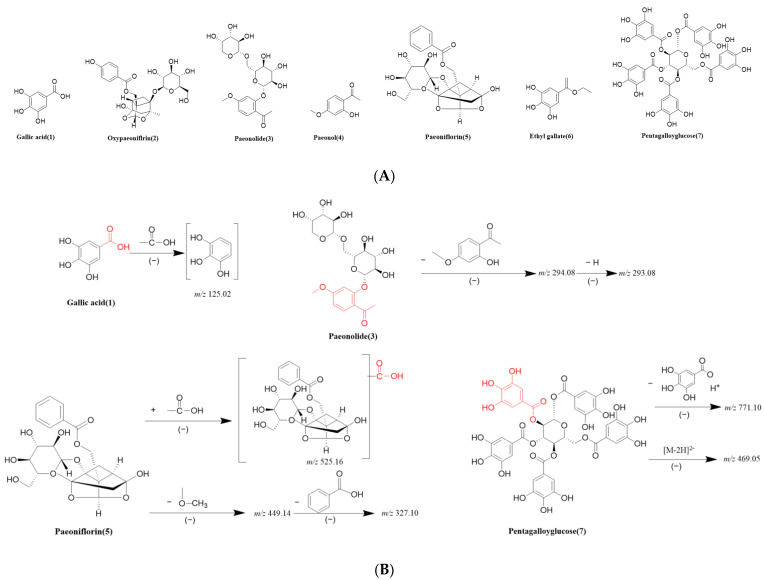
Chemical structures of the identified compounds (**A**), and the deduced fragmentation pathways of gallic acid, paeonolide, paeoniflorin, and pentagalloylglucose (**B**).

As indicated in the MS^2^ spectra in the positive and negative ion modes (Figures S1–S7), peak 1 showed the [M − H]^−^ ion at *m*/*z* 169.0137. The fragment ion at *m*/*z* 125.0236 was generated by the loss of a carboxyl, whose molecular formula was determined as C_7_H_6_O_5_, with a molecular mass of 170 Da. Peak 1 was identified as gallic acid, which was in accordance with the findings of a previous study [61]. Peak 2 displayed the [M − H]^−^ ion at *m*/*z* 495.1503; its molecular formula was definitely inferred as C_23_H_28_O_12_ from the [M + Na]^+^ ion at *m*/*z* 519.1485 and [M + K]^+^ ion at *m*/*z* 535.1225. Compared with the reference compound, it was deduced to be oxypaeoniflorin [62]. Peak 3 showed [M + Na]^+^ at *m*/*z* 483.1487 with a presumed molecular formula of C_20_H_28_O_12_. In the negative ion mode, there were fragment ions *m*/*z* 459.1499, indicating that compound 3 exhibited the [M − H]^−^ ion at *m*/*z* 459.1499, and the loss of paeonol (C_9_H_10_O_3_) gave rise to the fragment at *m*/*z* 293.0866, suggesting a paeonol derivative. The *m*/*z* 505.1558 is associated with the acquisition of a molecule of the carboxyl groups. Thus, this compound was tentatively assigned as paeonolide. Such identification of paeonolide was in agreement with a previous report [62]. Because the carbonyl and enoyl groups in paeonol readily form intramolecular hydrogen bonds, it is difficult for paeonol to negatively ionize under acidic conditions [50]. In the positive ion mode, compound 4 displayed the [M + H]^+^ ion at *m*/*z* 167.0706, which was observed to be stable and abundant with fewer fragment ions in the negative ion mode. Thus, peak 4 was characterized as paeonol. The molecular formula of peak 5 was designed as C_23_H_28_O_11_ from the [M + Na]^+^ at *m*/*z* 503.1547. The fragment ion at *m*/*z* 525.1609 might arise owing to the presence of a molecule of formic acid (45 Da) in the negative MS^2^ experiment since formic acid was used in the mobile phase. Taking the MS^2^ *m*/*z* 449.1446 and *m*/*z* 327.1078 into consideration, there was a consecutive loss of CH_3_O and the benzoyl group, resulting in the generation of the fragment ion [M − CH_3_O − C_7_H_6_O_2_]^−^ at *m*/*z* 327.1078. Based on the analysis above, peak 5 was unambiguously deduced as paeoniflorin. The identification of paeoniflorin was consistent with a previous report [61]. Compared with peak 1, the fragment ions at *m*/*z* 171.0287 and *m*/*z* 169.0136 were observed, which indicated that compound 6 was a derivative of gallic acid. Peak 6 exhibited [M − H]^−^ and [M + H]^+^ ions at *m*/*z* 197.0451 and 199.0610, corresponding to the molecular formula of C_9_H_10_O_5_. Thus, this compound was verified as ethyl gallate [62]. Peak 7 had a molecular ion peak at *m*/*z* 963 showing [M + Na]^+^ with a presumed relative molecular mass of 940, and the molecular ion peak obtained a hydrogen ion and lost a molecule of gallic acid, suggesting the chemical formula of C_41_H_32_O_26_. The doubly charged ion [M − 2H]^2−^ was readily detected in abundance under ESI source conditions. The detection of an ion at *m*/*z* 469.0517 is likely attributed to the presence of ion [M − 2H]^2−^ [46,62]. Thus, in negative ion mode, the fragment ions at *m*/*z* 939.1118 and *m*/*z* 469.0517 contributed to the loss of hydrogen and [M − 2H]^2−^ ion. After a comparison with previous research, peak 7 was confirmed to be pentagalloylglucose [48]. The chemical structures of seven compounds and the deduced fragment pathways of gallic acid, paeonolide, paeoniflorin, and pentagalloylglucose are displayed in Figure 8.

## 3. Materials and Methods

### 3.1. Reagents and Materials

The MC was provided by Heze Hua Rui Petroleum Co., Ltd., (Heze, China). The ethanol was supplied by Kaitong Chemical Reagent Co., Ltd., (Tianjin, China). The HPD-300 macroporous resin was provided by Beijing Solaibao Technology Co., Ltd., (Beijing, China), and the other resins were provided by LanXiao Technology New Material Co., Ltd., (Xi’an, China). The chromatographic methanol was obtained from Oceanpak Alexative Chemical Co., Ltd., (Gothenburg, Sweden).

### 3.2. Determination of Paeonol from MC

#### 3.2.1. Conditions of HPLC

The paeonol quantification was achieved by HPLC. Before the HPLC analysis, standard solutions of paeonol and the extract were filtered by 0.45 µm membranes. HPLC separation of the target analytes was performed with an Inertsil ODS-SP (5 µm, 4.6 × 250 mm (UP)) chromatographic column. Ultrapure water (A) and chromatographic methanol (B) were used as the mobile phase. Gradient elution conditions: 0–10 min, 40–60% B; 10–20 min, 60–80% B; 20–30 min, 80–100% B. The injection volume was 10 µL, the flow rate was 0.8 mL, and the column temperature was 30 °C. The detection wavelength was 274 nm.

#### 3.2.2. Plotting of Standard Curves

The paeonol standard was precisely weighed at 5.0 mg, placed in 10 mL volumetric flasks, dissolved with methanol, and shaken well to obtain a paeonol standard solution with a concentration of 0.5 mg/mL. A series of paeonol standard solutions (5, 10, 15, 20, 25 µg/mL) were prepared by removing 0.1, 0.2, 0.3, 0.4, and 0.5 mL of the paeonol standard solution with a pipette and adjusting the volume precisely to 10 mL with a methanol solution. The standard curve was drawn with the peak area y as the vertical coordinate and the paeonol concentration x as the horizontal coordinate. The regression equation of paeonol was y = 84,218x – 10,221, R^2^ = 0.9996.

### 3.3. Extraction Process of Paeonol from MC

#### 3.3.1. Single-Factor Experiments

Single-factor experiments were performed to determine the optimal range of each variable. After the MC was washed with deionized water, it was dried to a moisture content of 10% in a constant temperature drying oven at 45–50 °C, pulverized, and sieved (60–80 mesh). A quantity (1.0 g) of the MC powder was accurately weighed and mixed with 20 mL 60% ethanol solution before the ultrasonic-assisted extraction at 30 °C and a power of 300 W for 30 min using an ultrasound processor (Ultrasonic Instrument Co., Ltd., Kunshan, China). Then, the crude extract was filtered and analyzed by HPLC. The concentration of paeonol from the MC extract was determined in accordance with the regression equation in Section 3.2.2, and the yield of paeonol was calculated according to the following Equation (1):

Extraction yield of paeonol (mg/g) = CV/1000 M(1)

where C is the paeonol concentration (µg/mL); V is the extraction liquid volume (mL); and M is the sample weight (g).

According to the conditions above, the single factor experiments were conducted by changing one of the factors: ethanol concentration (from 20% to 100%), liquid-to-material ratio (from 10:1 to 30:1 mL/g), ultrasound time (from 10 to 50 min), ultrasound temperature (from 30 to 50 °C), and ultrasound power (from 240 to 480 W).

#### 3.3.2. Box–Behnken Design

On the basis of the single-factor experiments, a five-factor and three-level experiment design was carried out according to the BBD to optimize the extraction process of paeonol from MC.

### 3.4. Purification Process of Paeonol from MC

#### 3.4.1. Pretreatment of Macroporous Resin

The crude paeonol-enriched extract, obtained under the optimal conditions of the BBD, was concentrated by a rotary evaporator to a certain concentration and subjected to further purification using macroporous resin.

All the resins were pretreated referring to the method of previous study [26]. In brief, the resins were immersed overnight in anhydrous ethanol and flushed with deionized water to be ethanol-free, then immersed in a 5% NaOH solution (*w*/*v*) for 4 h and washed by deionized water until the pH of the filtrate reached neutral. They were then soaked in a 5% HCl solution (*w*/*v*) for 4 h and flushed with deionized water to make them pH value-neutral before the experiments.

#### 3.4.2. Screening of Macroporous Resins

The MC crude extract was dissolved with deionized water, and the concentration of paeonol was 0.8 mg/mL. A quantity (1.0 g) of the different types of macroporous resins (LSA-900C, HPD-100, HPD-300, AB-8, S-8, D-101, NKA-9, HPD-826) that had been pre-treated were mixed with 25 mL MC crude extract (0.8 mg/mL) in 50 mL conical flasks. All the flasks were put in a constant temperature shaker (120 rpm) and shaken for 24 h at 25 °C. Following the absorption equilibrium, the resins were filtered, and the content of paeonol in the solutions was determined by HPLC after adsorption equilibrium.

After adsorption saturation, the different types of macroporous resins were washed with distilled water until the surface of resin had no residual MC extract, and the surface water of the macroporous resins was sucked up with filter paper. Subsequently, the resins were desorbed with 25 mL of 50% aqueous ethanol solution in a constant temperature water bath shaker (120 rpm) for 24 h at 25 °C. After desorption, the concentration of paeonol was measured. The adsorption capacity, adsorption ratio, desorption ratio, and recovery ratio were calculated according to the following Equations (2)–(5):

Adsorption capacity (mg/g)

Q_e_ = (C_0_ − C_1_) V_0_/M (2)

Adsorption ratio (%)

Q_d_ = [(C_0_ − C_1_)/C_0_] × 100 (3)

Desorption ratio (%)

D = [C_2_V_2_/(C_0_V_0_ − C_1_V_1_)] × 100 (4)

Recovery ratio (%)

R = (C_2_V_2_/C_0_V_0_) × 100 (5)

where Q_e_ (mg/g) is the adsorption capacity at adsorption equilibrium; C_0_ and C_1_ (mg/mL) are the initial and equilibrium concentrations of paeonol in the solutions, respectively; V_0_ (mL) is the volume of the crude extract; M (g) is the weight of the resin; V_1_ (mL) is the volume of the crude extract after adsorption; C_2_ (mg/mL) is the concentration of paeonol in the desorption solution; V_2_ (mL) is the volume of the desorption solution; and R (%) is the recovery ratio.

#### 3.4.3. Static Adsorption and Desorption Tests

The adsorption and desorption static experiments were carried out as follows. A quantity (1.0 g) of pretreated HPD-300 macroporous resin and 25 mL of the sample solution with a concentration of 0.8 mg/mL for paeonol were added into conical flasks (50 mL), and the flasks were continually shaken at 120 rpm for 12 h at a constant temperature (25 °C). During this period, 100 µL of the sample solution was taken every hour, and the paeonol concentrations were determined by HPLC. The adsorption ratios were calculated, and the adsorption kinetic curve was plotted.

When adsorption equilibriums were reached, the macroporous resins were washed with deionized water. A mixture of 25 mL 50% ethanol solution and macroporous resin after adsorption equilibrium were shaken at 120 rpm at 25 °C for 12 h after adsorption equilibrium. The paeonol concentration was measured in 100 µL of the supernatant every hour. The desorption ratios of HPD-300 macroporous resin were measured, and the desorption kinetic curve was plotted. Under the same pH conditions, seven crude MC solutions with different initial paeonol concentrations (0.1, 0.2, 0.4, 0.6, 0.8, 1.0, and 1.4 mg/mL) were employed to investigate the effect of the paeonol concentration on the adsorption process. The effect of pH on static absorption was researched by adjusting the pH from 2 to 8. Static desorption tests were developed with an ethanol eluent at different concentrations in a range of 20–100% (*v*/*v*).

#### 3.4.4. Dynamic Adsorption and Desorption Tests

Dynamic adsorption and desorption tests were carried out on a glass column (0.6 cm × 50 cm) with pretreated HPD-300 macroporous resin (10.0 g). The absorption process was performed by loading a paeonol solution according to the optimal conditions and changing the sample flow rate (1.0, 1.5, 2.0, 2.5, and 3.0 mL/min). A fraction collector was used for collecting the eluent solution. Approximately 10 mL eluent was collected in each tube, and every two tubes were combined to determine the concentration of paeonol by HPLC. The dynamic adsorption curves were plotted at the five flow rates. The optimal sample volume and flow rate were determined according to the dynamic adsorption curve.

After adsorption equilibrium, the adsorption column was firstly washed with deionized water, and then desorbed with ethanol solution. The elution process was performed with a 70% ethanol solution and at the elution flow rates of 1.0, 1.5, 2.0, 2.5, and 3.0 mL/min. The effluent was analyzed by HPLC, and the dynamic desorption curve was plotted. The optimal eluent volume and elution flow rate were determined according to the dynamic desorption curve.

The effluent containing paeonol was collected, further concentrated, and dried under a vacuum to calculate the purity of the paeonol.

### 3.5. Identification of MC Extract

#### 3.5.1. Preparation of Sample Solution

Purification of the MC extract was carried out according to the optimal purification conditions. The effluent containing paeonol was concentrated in a rotary evaporator at 45 °C to obtain the MC concentrated liquid. It was then diluted with a 50% ethanol (HPLC grade) aqueous solution and filtered through 0.22 μm membrane filters for UPLC-Q-TOF-MS/MS analysis.

#### 3.5.2. Conditions of UPLC-Q-TOF-MS/MS

The UPLC analysis was performed with a Nexera UPLC system (Shimadzu, Kyoto, Japan) equipped with an Agilent ZORBAX Eclipse Plus C18 (4.6 × 250 mm, 5 μm) chromatographic column and an autosampler coupled with a PDA detector.

The mobile phases consisted of 0.1% (*v*/*v*) formic acid in water (A) and methanol (B), and the gradient program was configured as follows: 0–10 min, 15–20% B; 10–20 min, 20–30% B; 20–23 min, 30–32% B; 23–30 min, 32–35%; 30–35 min, 35%; 35–40 min, 35–40%; 40–50 min, 40–50%; 50–60 min, 50–60%; 60–80 min, 60–80%. The wavelength of detection was 254 nm, and the flow rate was 0.8 mL/min with the column temperature at 30 °C and the injection volume as 10 μL. The mass spectrometry analysis conditions were as follows: ion source, ESI; capillary voltage, +4.0 kV/−3.0 kV; atomization gas flow rate, 3.0 L/min; dryer temperature, 250 °C; nebulizer gas (N_2_) pressure, 2 × 10^5^ Pa; drying gas flow rate, 10.0 L/min; scanning range of *m*/*z* 50–1500.

### 3.6. Statistical Analysis

The data were presented as mean ± standard deviation (SD) and evaluated by one-way analysis of variance, followed by Duncan’s multiple-range tests. The difference was considered to be statistically significant if *p* < 0.05. All the statistical analyses were conducted by SPSS 26.0 (Chicago, IL, USA). All the charts were created by GraphPad Prism 9.0 (GraphPad Software Inc, San Diego, CA, USA).

## 4. Conclusions

In this study, paeonol was extracted from MC using an ultrasound-assisted ethanol extraction process. Based on the single-factor experiments and BBD, the optimum extraction conditions were obtained as follows: liquid-to-material ratio, 21:1 mL/g; ethanol concentration, 62%; ultrasonic time, 31 min; ultrasonic temperature, 36 °C; and ultrasonic power, 420 W. Under these conditions, the yield of paeonol reached 14.01 mg/g. The optimal purification conditions were gained using HPD-300 macroporous resin: the sample concentration, 1.0 mg/mL; the sample pH, 4; the ethanol concentration of the eluent, 70%; the loading flow rate, 2.0 mL/min; the sample volume, 180 mL; the elution flow rate, 1.5 mL/min; and the elution volume, 140 mL. The purity of paeonol reached 41.40% (5.97 times higher than that of the crude extract), indicating that macroporous resin adsorption was an effective purification method. Seven compounds were identified by qualitative analysis of the main components from the MC extract, including gallic acid, oxypaeoniflorin, paeonolide, paeonol, paeoniflorin, ethyl gallate, and pentagalloylglucose. This indicates that the MC extract was not only rich in phenolic compounds, but also rich in monoterpene glycosides. How to comprehensively utilize these active components while extracting and purifying paeonol remains to be further researched. These results will provide a theoretical basis for deep processing and utilization of MC.

## Figures and Tables

**Table 1:** Response surface test factors and horizontal design.

Factors	Level		
	−1	0	1
Liquid-to-material ratio/mL/g	15	20	25
Ethanol concentration/%	40	60	80
Ultrasonic time/min	20	30	40
Ultrasonic temperature/°C	30	35	40
Ultrasonic power/W	360	420	480

**Table 2:** Experimental design and response value of Box–Behnken.

Run	A	B	C	D	E	Extraction Yield (mg/g)
1	15	40	30	35	420	7.43 ± 0.52
2	25	40	30	35	420	8.52 ± 0.44
3	15	80	30	35	420	8.27 ± 0.51
4	25	80	30	35	420	9.41 ± 0.14
5	20	60	20	30	420	9.63 ± 0.02
6	20	60	40	30	420	9.86 ± 0.08
7	20	60	20	40	420	8.93 ± 0.56
8	20	60	40	40	420	10.16 ± 0.41
9	20	60	30	35	360	7.39 ± 0.23
10	20	80	30	35	360	8.26 ± 0.44
11	20	40	30	35	480	8.23 ± 0.17
12	20	80	30	35	480	8.81 ± 0.21
13	15	60	20	35	420	8.43 ± 0.48
14	25	60	20	35	420	9.55 ± 0.19
15	15	60	40	35	420	8.84 ± 0.64
16	25	60	40	35	420	10.43 ± 0.71
17	20	60	30	30	360	8.36 ± 0.23
18	20	60	30	40	360	8.29 ± 0.12
19	20	60	30	30	480	8.82 ± 0.16
20	20	60	30	40	480	9.66 ± 0.05
21	20	40	20	35	420	8.32 ± 0.19
22	20	80	20	35	420	10.33 ± 0.22
23	20	40	40	35	420	9.84 ± 0.87
24	20	80	40	35	420	10.01 ± 0.45
25	15	60	30	30	420	7.98 ± 0.33
26	25	60	30	30	420	9.34 ± 0.06
27	15	60	30	40	420	8.54 ± 0.45
28	25	60	30	40	420	10.19 ± 0.10
29	20	60	20	35	360	8.27 ± 0.25
30	20	60	40	35	360	8.53 ± 0.33
31	20	60	20	35	480	9.32 ± 0.47
32	20	60	40	35	480	9.38 ± 0.11
33	15	60	20	35	360	7.87 ± 0.80
34	25	60	40	35	360	8.05 ± 0.44
35	15	60	30	35	480	7.22 ± 0.56
36	25	60	30	35	480	9.84 ± 0.14
37	20	40	30	30	420	9.23 ± 0.73
38	20	80	30	30	420	8.53 ± 0.40
39	20	40	30	40	420	9.09 ± 0.44
40	20	80	30	40	420	10.78 ± 0.02
41	20	60	30	35	420	13.67 ± 0.01
42	20	60	30	35	420	12.98 ± 0.09
43	20	60	30	35	420	12.18 ± 0.04
44	20	60	30	35	420	13.01 ± 0.11
45	20	60	30	35	420	13.74 ± 0.08
46	20	60	30	35	420	12.22 ± 0.16

**Table 3:** ANOVA of Box–Behnken.

ANOVA Source	Sum of Squares	df	Mean Square	F-Value	*p*-Value
Model	112.37	20	5.62	32.81	<0.0001 ***
A	7.09	1	7.09	41.40	<0.0001 ***
B	2.52	1	2.52	14.72	0.0008 ***
C	1.36	1	1.36	7.96	0.0092 ***
D	0.8978	1	0.8978	5.24	0.0307 *
E	2.15	1	2.15	12.53	0.0016 **
AB	0.0006	1	0.0006	0.0037	0.9523
AC	0.0552	1	0.0552	0.3225	0.5752
AD	0.0090	1	0.0090	0.0527	0.8203
AE	1.49	1	1.49	8.69	0.0068 **
BC	0.8464	1	0.8464	4.94	0.0355 *
BD	1.43	1	1.43	8.34	0.0079 **
BE	0.0210	1	0.0210	0.1228	0.7290
CD	0.2500	1	0.2500	1.46	0.2382
CE	0.0900	1	0.0900	0.5256	0.4752
DE	0.2070	1	0.2070	1.21	0.2820
A^2^	46.00	1	46.00	268.66	<0.0001 ***
B^2^	37.61	1	37.61	219.64	<0.0001 ***
C^2^	17.78	1	17.78	103.87	<0.0001 ***
D^2^	24.41	1	24.41	142.58	<0.0001 ***
E^2^	56.97	1	56.97	332.74	<0.0001 ***
Residual	4.28	25	0.1712		
Lack of fit	2.01	20	0.1005	0.2212	0.9936
Pure error	2.27	5	0.4542		
Cor total	116.65	45			
	R^2^ = 0.9633 R^2^_Adj_ = 0.9339 C.V.% = 4.37				

Notes: Level of significance: * *p* < 0.05, ** *p* < 0.01, *** *p* < 0.001.

**Table 4:** UPLC-Q-TOF-MS/MS analysis of MC extract.

No.	Retention Time (min)	Compound	Formula	M.W.	Select Ions	Quasimol-Ecular Ion (*m*/*z*)	Fragment Ions (*m*/*z*)
1	7.48	Gallic acid	C_7_H_6_O_5_	170	[M + H]^+^	171	127
	7.43				[M − H]^−^	169	125
2	22.80	Oxypaeoniflorin	C_23_H_28_O_12_	496	[M + Na]^+^, [M + K]^+^	519	332, 275, 189
	23.48				[M − H]^−^	495	137
3	29.46	Paeonolide	C_20_H_28_O_12_	460	[M + Na]^+^, [C_9_H_10_O_3_ + H]^+^	483	167
	29.53				[M − H]^−^, [M + HCOO]^−^, [M − C_9_H_10_O_3_ − H]^−^	505	459, 293
4	32.30	Paeonol	C_9_H_10_O_3_	166	[M + H]^+^	167	97
	32.63						165, 125
5	37.45	Paeoniflorin	C_23_H_28_O_11_	480	[M + Na]^+^	503	179, 280
	37.59				[M + HCOO]^−^	525	327, 177, 195
6	39.44	Ethyl gallate	C_9_H_10_O_5_	198	[M + H]^+^	199	171, 109
	39.34				[M − H]^−^, [M − 2H]^2−^	197	317, 169
7	57.77	Pentagalloylglucose	C_41_H_32_O_26_	940	[M + Na]^+^, [M – C_7_H_6_O_5_]^+^	963	771, 171, 127
	57.70				[M – H]^−^, [M – 2H]^2−^	939	469, 169, 125

## Data Availability

All the data are available within the article and Supplementary Materials.

## Supplementary Materials

The following supporting information can be downloaded at: https://www.mdpi.com/article/10.3390/molecules29030622/s1, Table S1: Chemical and physical properties of different macroporous resins; Figure S1: The mass spectrum of peak 1: positive ion mode (a); negative ion mode (b); Figure S2: The mass spectrum of peak 2: positive ion mode (a); negative ion mode (b); Figure S3: The mass spectrum of peak 3: positive ion mode (a); negative ion mode (b); Figure S4: The mass spectrum of peak 4: positive ion mode (a); negative ion mode (b); Figure S5: The mass spectrum of peak 5: positive ion mode (a); negative ion mode (b); Figure S6: The mass spectrum of peak 6: positive ion mode (a); negative ion mode (b); Figure S7. The mass spectrum of peak 7: positive ion mode (a); negative ion mode (b).
