# Supplementary material for: Ultrasound-Assisted Extraction of Paeonol from Moutan Cortex: Purification and Component Identification of Extract
Source: Molecules. 2024 Jan 28;29(3):622. doi: 10.3390/molecules29030622 (PMC10856641; doi:10.3390/molecules29030622)
Supplement: Supplementary file 1 [file molecules-29-00622-s001.zip › molecules-2798650-supplementary.pdf]

Supplementary materials

Table S1. Chemical and physical properties of different macroporous resins.

Type of resin	Polarity	Specific surface area（m^2/g）	Pore diameters（nm）
HPD-300	nonpolar	800-870	27
LSA-900C	nonpolar	>500	12
D-101	nonpolar	⩾550	13-14
HPD-100	nonpolar	＞550	8.5-9.0
AB-8	weak polar	480-520	13-14
HPD-826	polar	500-600	9.0-10
NKA-9	polar	270	15-16.5
S-8	polar	100-120	28-30

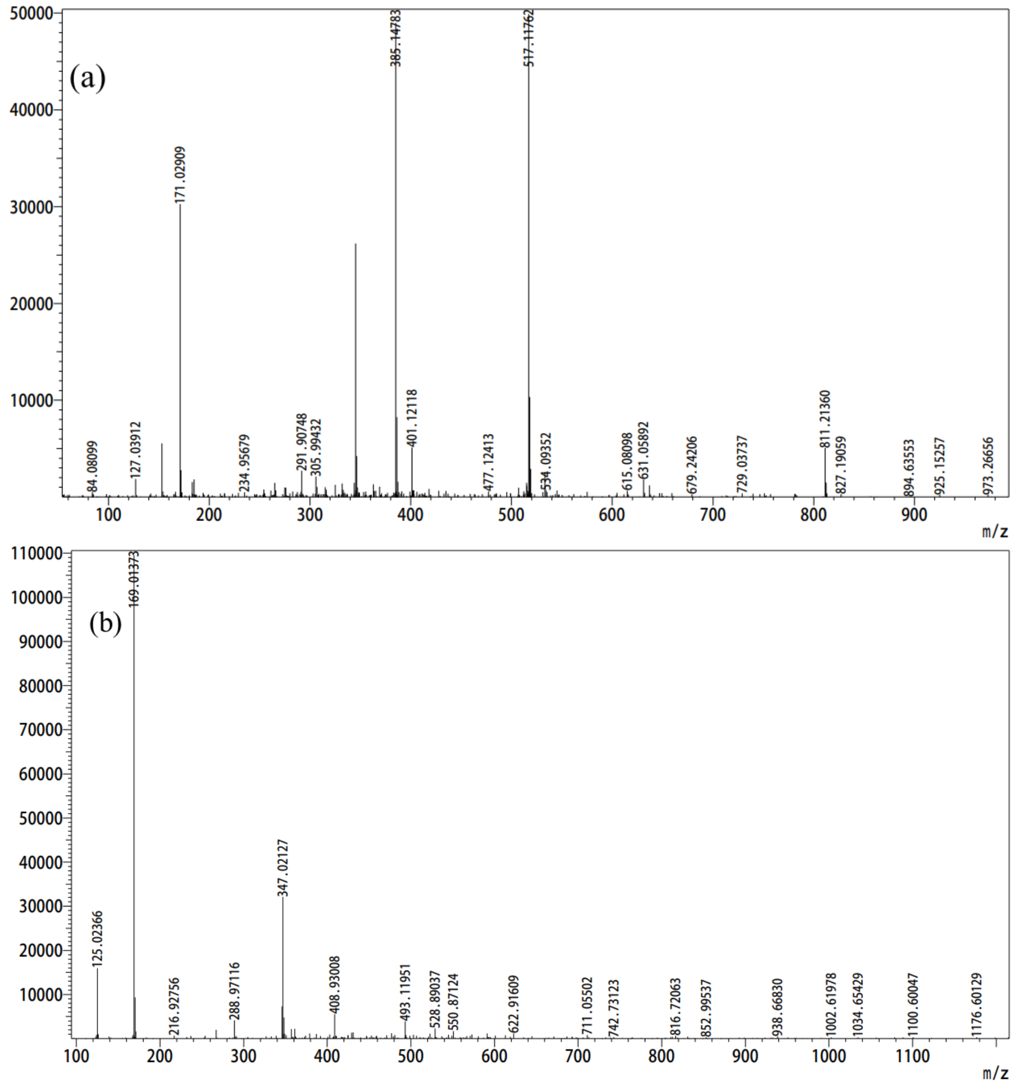

Figure S1. The mass spectrum of peak 1: positive ion mode (a); negative ion mode (b).

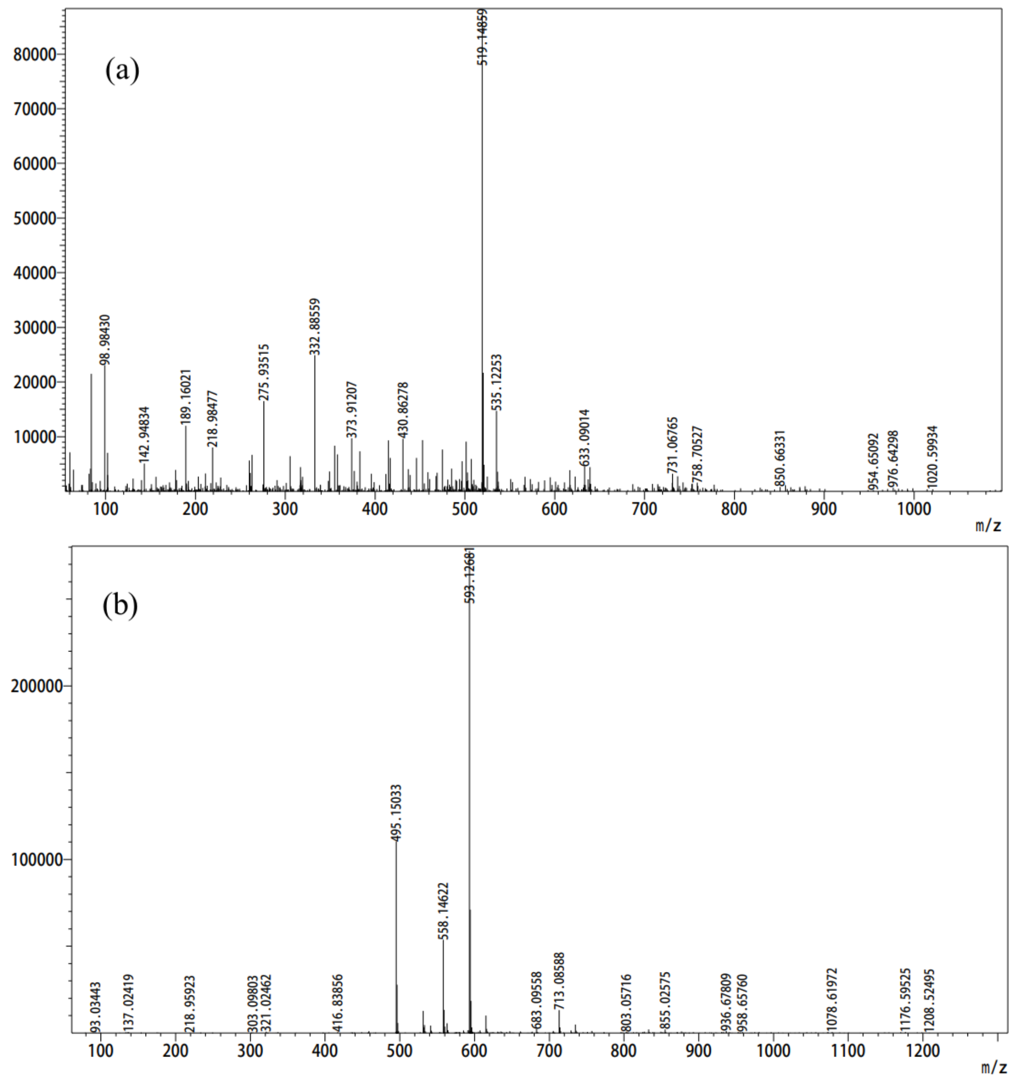

Figure S2. The mass spectrum of peak 2: positive ion mode (a); negative ion mode (b).

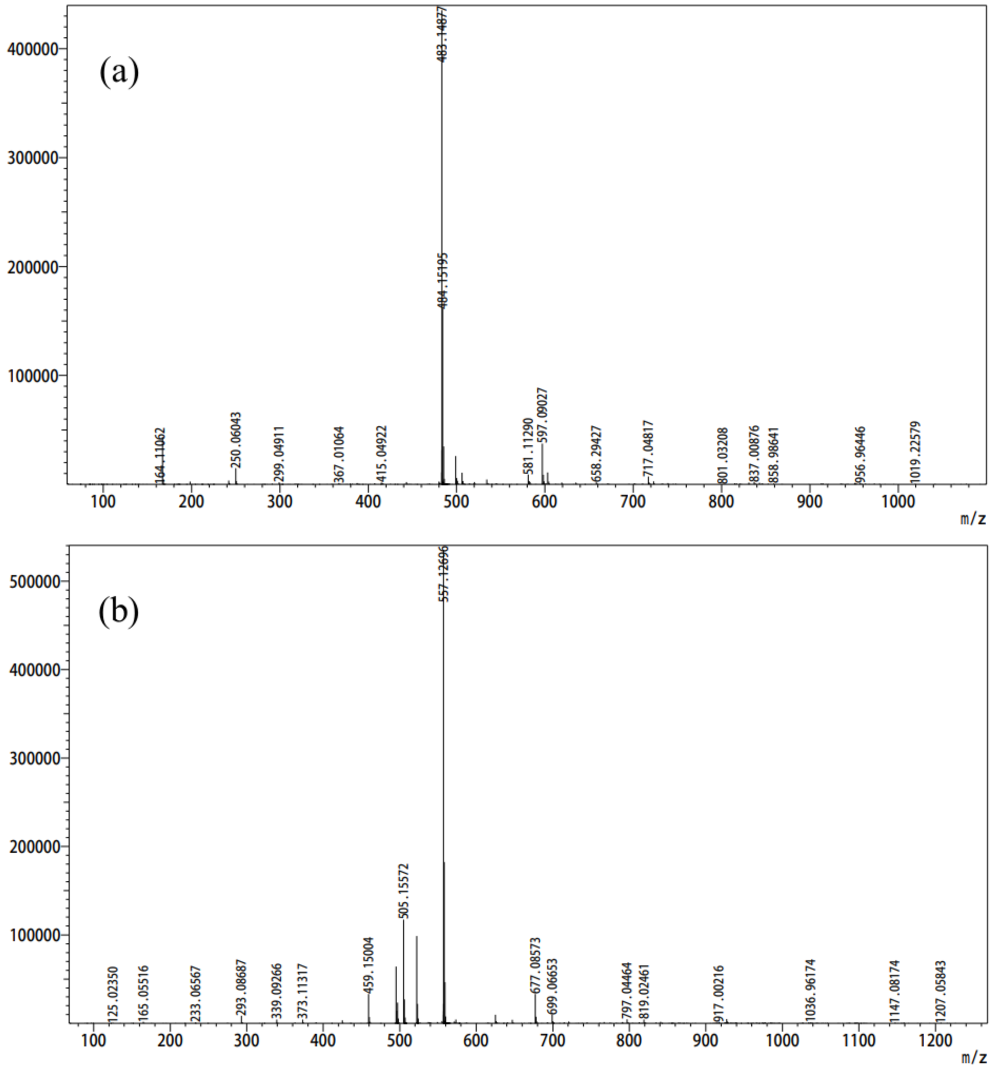

Figure S3. The mass spectrum of peak 3: positive ion mode (a); negative ion mode (b).

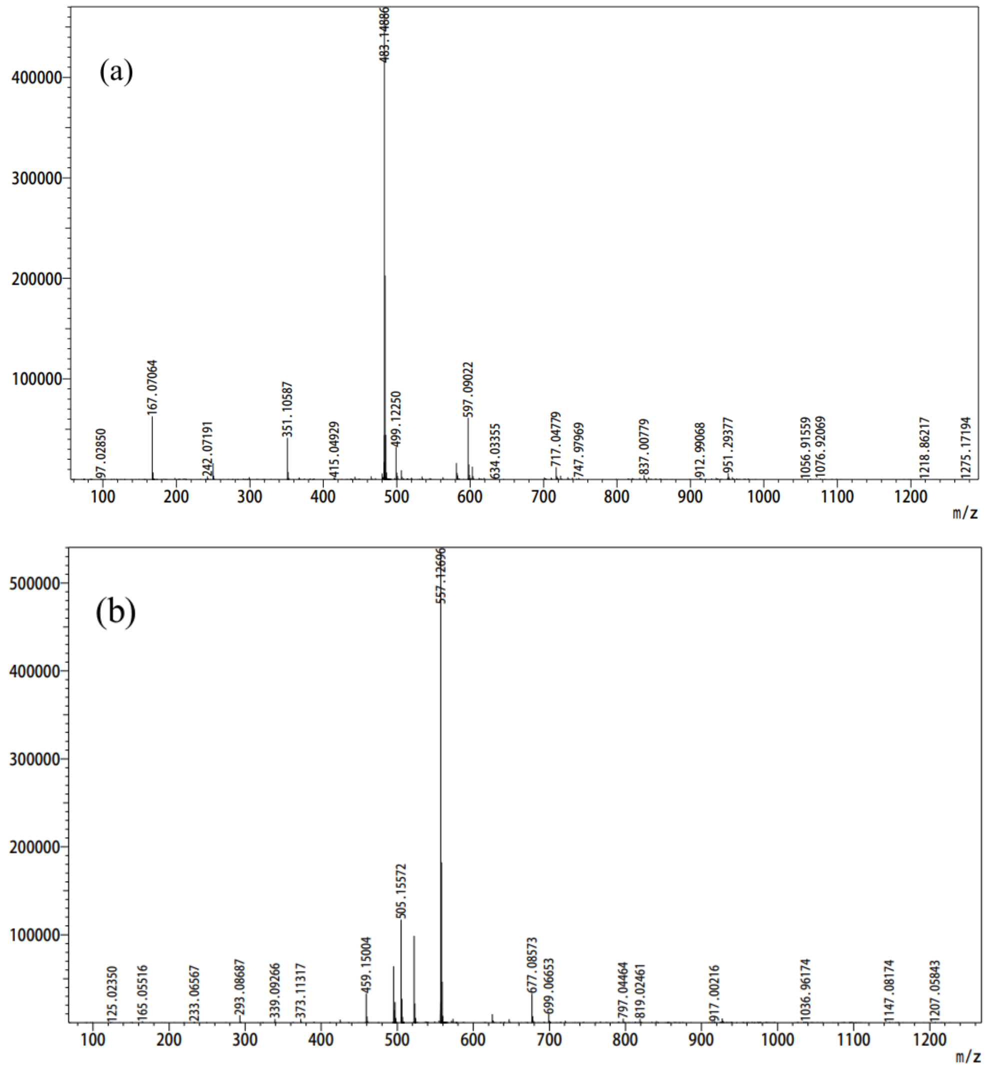

Figure S4. The mass spectrum of peak 4: positive ion mode (a); negative ion mode (b).

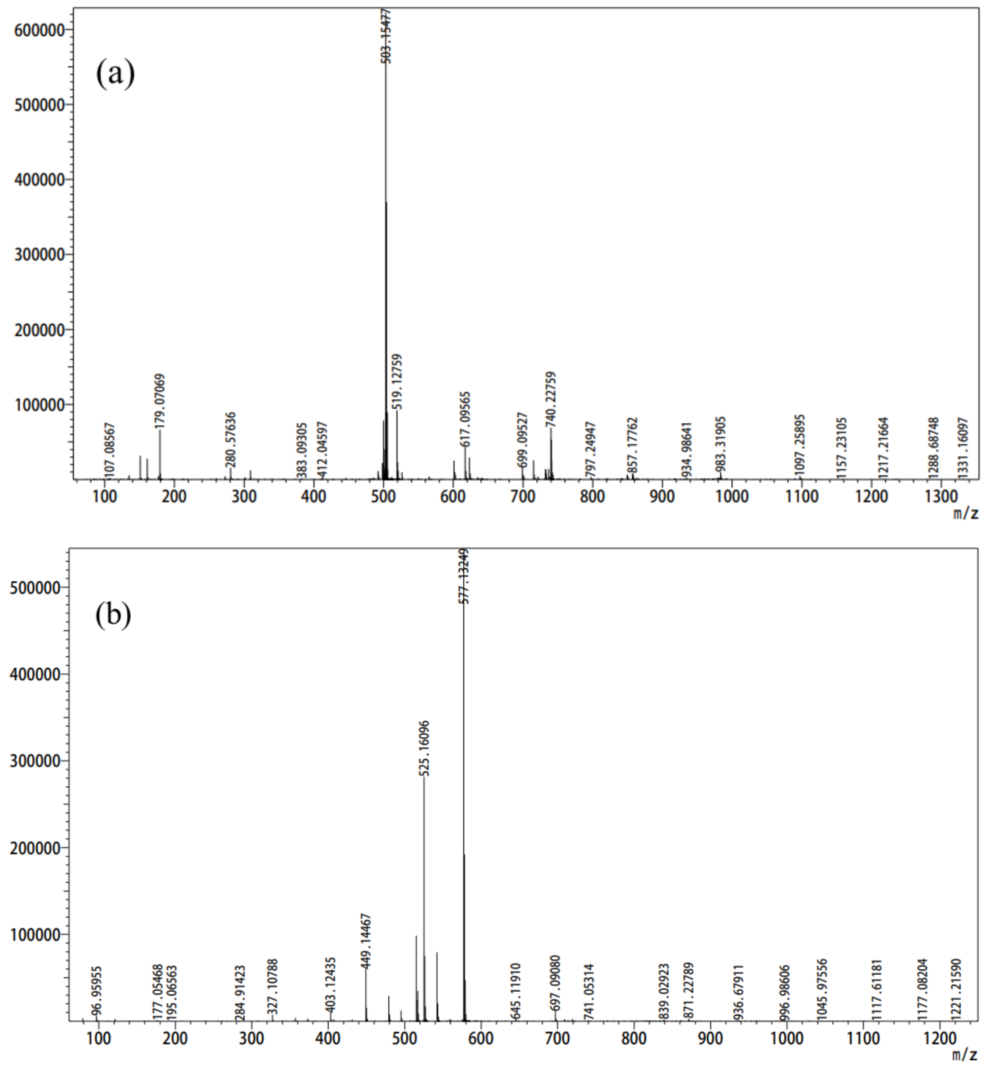

Figure S5. The mass spectrum of peak 5: positive ion mode (a); negative ion mode (b).

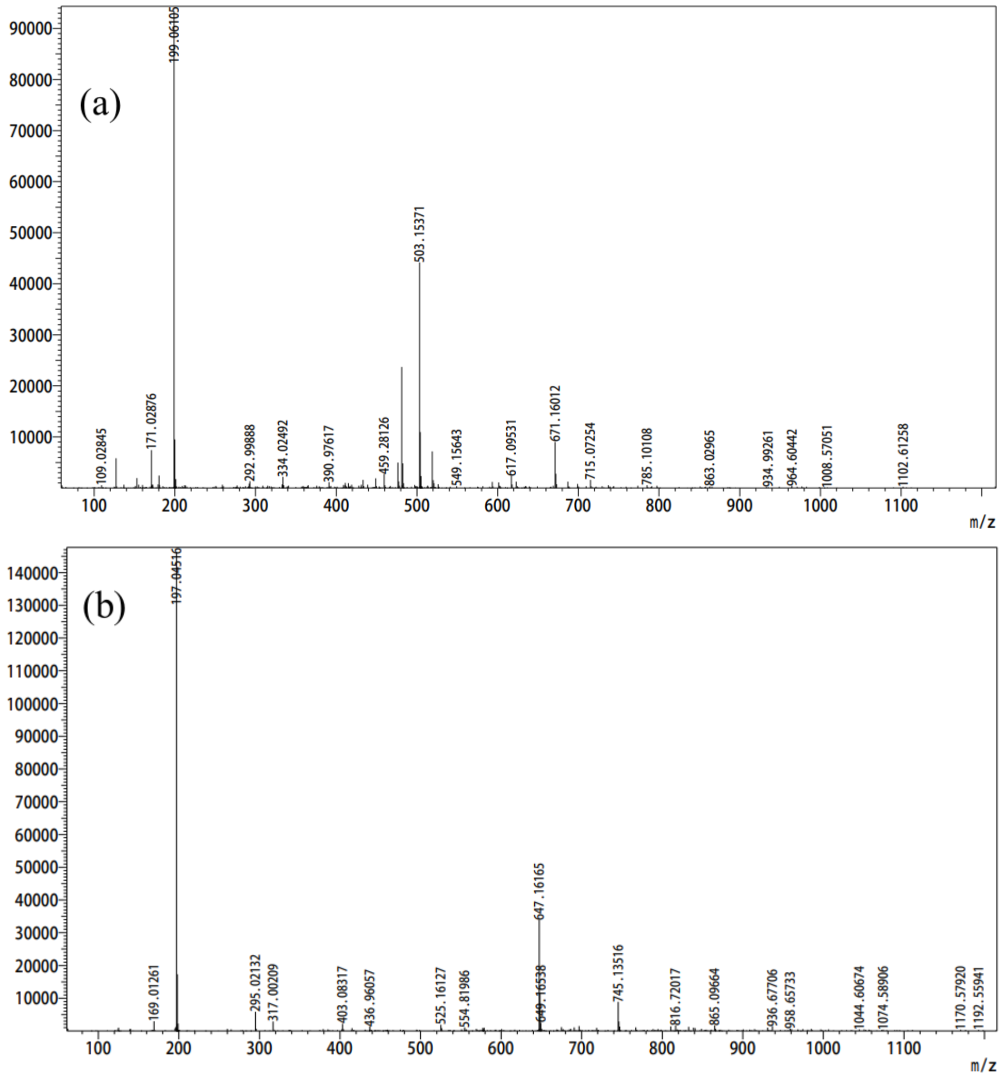

Figure S6. The mass spectrum of peak 6: positive ion mode (a); negative ion mode (b).

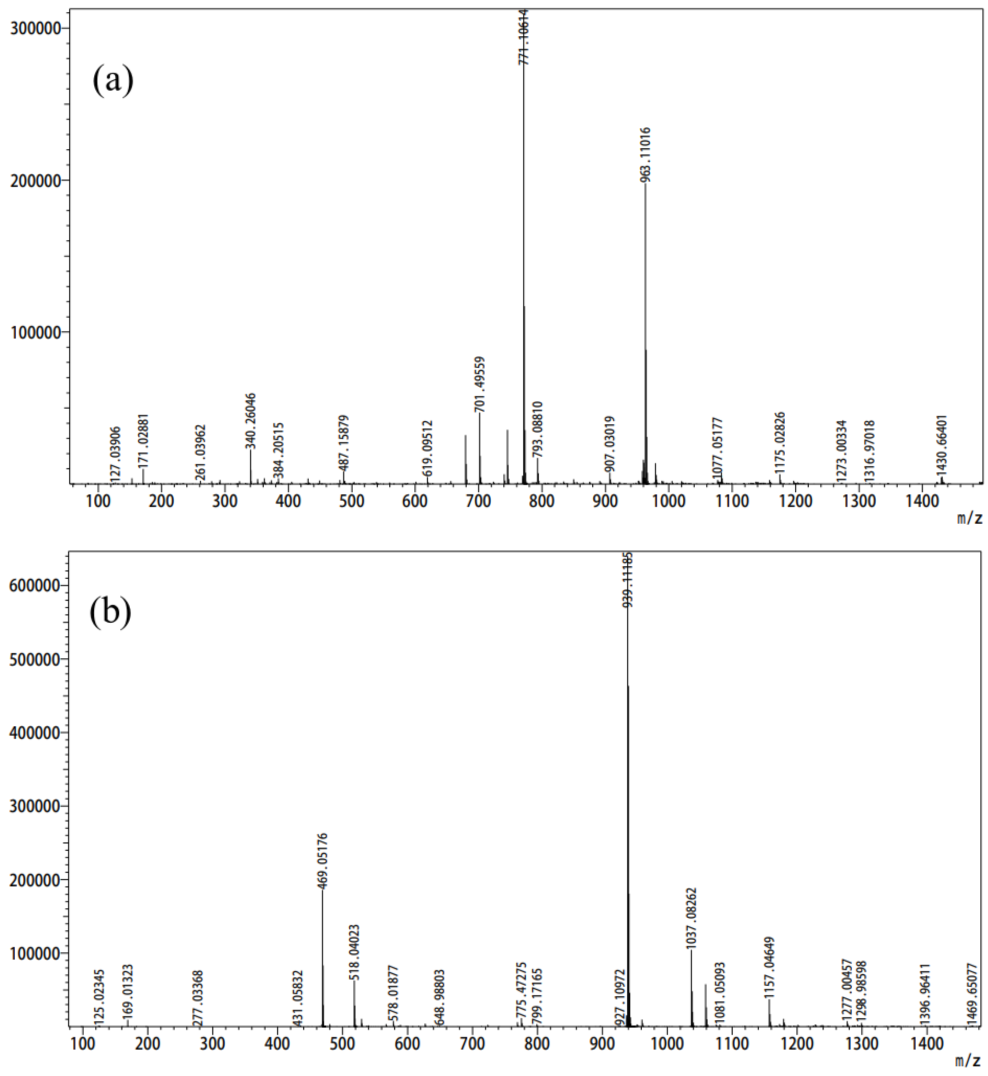

Figure S7. The mass spectrum of peak 7: positive ion mode (a); negative ion mode (b).
